# Missed opportunities for institutional delivery and associated factors among urban resident pregnant women in South Tigray Zone, Ethiopia: a community-based follow-up study

**DOI:** 10.3402/gha.v8.28082

**Published:** 2015-09-09

**Authors:** Hinsermu Bayu, Girmastion Fisseha, Amlaku Mulat, Gebre Yitayih, Mengistu Wolday

**Affiliations:** 1 Department of Midwifery, College of Health Sciences, Mekelle University, Mekelle, Ethiopia; 2 School of Public Health, College of Health Sciences, Mekelle University, Mekelle, Ethiopia; 3 Department of Nursing, College of Health Sciences, Mekelle University, Mekelle, Ethiopia

**Keywords:** missed, opportunities, institutional, delivery, ethiopia

## Abstract

**Background:**

Every pregnant woman is considered to be at risk and some risks may not always be foreseeable or detectable. Therefore, the presence of a skilled birth attendant at every delivery is considered to be the most critical intervention in reducing maternal mortality and morbidity. In Ethiopia, the proportion of births attended by skilled personnel in urban settings can be as low as 10%. Therefore, the main purpose of this research was to identify factors affecting unplanned home delivery in urban settings, where there is relatively good access in principle to modern healthcare institutions.

**Design:**

A community-based follow-up study was conducted from 17 January 2014 to 30 August 2014, among second- and third-trimester pregnant women who had planned for institutional delivery in South Tigray Zone. A systematic sampling technique was used to get a total of 522 study participants. A pre-tested and structured questionnaire was used to collect relevant data. Bivariate and multivariate data analyses were performed using SPSS version 16.0.

**Results:**

The study revealed that among 465 pregnant women who planned for institutional delivery, 134 (28.8%) opted out and delivered at their home (missed opportunity). Single women (AOR 2.34, 95% CI 1.17–4.68), illiterate mothers (AOR 6.14, 95% CI 2.20–17.2), absence of antenatal clinic visit for indexed pregnancy (AOR 3.11, 95% CI 1.72–5.61), absence of obstetric complications during the index pregnancy (AOR 2.96, 95% CI 1.47–5.97), poor autonomy (AOR 2.11, 95% CI 1.27–3.49), and absence of birth preparedness and complication readiness (AOR 3.83, 95% CI 2.19–6.70) were significant predictors of unplanned home delivery.

**Conclusions:**

A significant proportion of pregnant women missed the opportunity of modern delivery assistance. Educational status, antenatal care status, lack of obstetric complications, poor autonomy, and lack of birth preparedness and complication readiness were among the important predictors of unplanned home delivery.

Globally, maternal mortality ratios (MMRs) have declined by 45% between 1990 and 2013. Despite this recent progress, there were still an estimated 289,000 maternal deaths annually as of 2013. Developing countries account for 99% (286,000) of global maternal deaths, with the sub-Saharan African region accounting for 62% (179,000) followed by Southern Asia 23.9% (69,000) (1). Although sub-Saharan Africa's contribution to the global maternal death burden has reduced from 87% in 2008 to 62% in 2013, it remains as the only developing region with very high MMRs. Compared to other regions, women in sub-Saharan Africa face a higher lifetime risk of death (1 in 31) due to pregnancy and child-birth-related complication (1, 2). This implies that every day a significant number of reproductive age group women die mainly due to preventable pregnancy and child-birth-related causes.

MMR in Ethiopia is still among the highest in the world, and Ethiopia is one of 10 countries that contributed 60% of the global maternal death burden in 2010 (3). Various national estimates for MMR in Ethiopia in recent years have been summarised, mostly in the region of around 400 per 100,000, though the EDHS in 2011 made a higher estimate (4). Millennium Development Goal 5 (MDG 5) aims to improve maternal health by reducing MMR by at least 75% between 1990 and 2015. All MDG regions of the world have experienced considerable reductions in maternal mortality, but the average annual percentage decline of MMR in sub-Saharan Africa, to which Ethiopia contributes considerably, is only 1.7%. This is very much lower than the MDG target of 5.5% (1, 2).

The risk assessment approach to antenatal care (ANC) currently being used is outdated and is being replaced by disease detection as one component of focused ANC (5). So the best way to assure a safe and successful delivery outcome remains to be ensuring the presence of a skilled birth attendant at every child birth, which needs to be prioritised. This is also one of the indicators used in assessing MDG5 (6).

Despite the need for skilled birth attendance, the proportion of births attended by skilled personnel in Ethiopia is only 10% nationally, but 49% in urban areas where women have better access to services (7). Many researchers have identified a lack of health institutions, transportation, and women's motivation as main factors forcing women to choose home delivery (6, 7). However, a significant number of women in urban settings, where there is relatively good access to health institutions and transportation, continue to deliver at home. Thus, the main purpose of this research was to identify factors affecting unplanned home delivery in urban contexts, where women had planned for institutional delivery but not fulfilled their intentions.

## Methods

A community-based follow-up study was conducted in South Tigray Zone from 17 January 2014 to 30 August 2014 among urban resident pregnant women in their second or third trimesters. Alamata, the capital city of the zone, is located 560 km north-east of Addis Ababa, the capital city of Ethiopia. Each town (Alamata, Mehoni, and Maichew) in the zone has at least one health institution that can provide maternal and child health services.

The potential study population comprised all pregnant women who were in their second or third trimester of pregnancy at the time of survey. Respondents were identified using a systematic sampling technique from a list of second- or third-trimester pregnant women obtained from health extension workers in each kebele of the respective towns. Proportional sample size allocation was used to select a representative sample from the three towns. The sample size was determined using a single population proportion formula considering the following assumptions: magnitude of missed opportunity of institutional delivery in urban context 50%, 4.5% level of significance (*α*=0.045). The final sample size was adjusted for a non-response rate of 10% and the total sample arrived at was 522.

The outcome variable, missed opportunity of institutional delivery, identified the pregnant women who planned for institutional delivery but unfortunately ended up with home child birth. Data were collected through face-to-face interviews using a structured and pre-tested questionnaire while conducting house-to-house survey. Final-year diploma midwifery students who were capable of taking obstetric histories collected the data. Two midwives from the health institution supervised the data-collection process. The data collection had two phases. In phase I, pregnant women were interviewed to assess their socio-demographic profile, preference about place of delivery (home or health institution) and some factors associated with their planned place of delivery. In phase II, the pregnant women who had been interviewed in phase I were revisited after 6 months to determine their actual place of delivery and the associated factors for their actual place of delivery (home or health institution).

Data analysis was performed using SPSS version 16.0. Variables reaching a *p*-value of 0.2 on bivariate analysis were included in multiple logistic regression analysis and *p*-values of <0.05 were considered significant. The degree of association between the independent and dependent variables was analysed using odds ratios with 95% confidence intervals.

Ethical clearance was obtained from the Institutional Review Board (IRB) of Mekelle University, College of Health Sciences. A formal letter of cooperation was sent to South Tigray Zone Health Bureau and a formal letter of permission was obtained. Finally, written informed consent was obtained from each pregnant woman.

## Results

### Socio-demographic characteristics

Among the 522 sampled pregnant women in their second or third trimester, 465 responded to the questionnaire, giving a response rate of 89%. The mean age of the study participants was 28.7 years (SD 6.4). The majority of women were Tigrean (384, 82.6%), married (339, 72.9%), and orthodox Christian (333, 71.6%). Two hundred and ninety seven (63.9%) of the pregnant women had attended at least primary education (Table 1).

**Table 1:** Socio-demographic characteristics of mothers (*n*=465) who were in their second and third trimesters of pregnancy in South Tigray Zone towns, November, 2014

Characteristics	Frequency (%)
Age of mothers during the interview (mean, SD 28.7±6.4 SD)	
15–19	33 (7.1)
20–24	109 (23.4)
25–29	136 (29.2)
>30	187 (40.2)
Marital status	
Single	68 (14.6)
Married	339 (72.9)
Divorced	20 (4.3)
Widowed	38 (8.2)
Religion	
Muslim	103 (22.2)
Orthodox	333 (71.6)
Protestant	19 (4.1)
Other*	10 (2.2)
Educational status	
No formal education	168 (36.1)
Primary education	143 (30.8)
Secondary and high school	78 (16.8)
Collage and above	76 (16.3)
Ethnicity	
Tigre	384 (82.6)
Amhara	64 (13.8)
Other**	17 (3.7)
Occupation	
Housewife	263 (56.6)
Self-employed	66 (14.2)
Government employee	80 (17.2)
Students/daily workers	56 (12)

* Other=Catholic, Seventh Day Adventist;

** Other=Gurage, Agawu, Afar.

### Obstetric characteristics

For 118 (25.4%) of the women, the index pregnancy was their first, while 263 (56.6%) of them had experienced pregnancy 2–4 times. Four hundred and eleven (88.4%) of the women had faced at least one obstetric problem during the index pregnancy or during labour. Among the respondents, 345 (74.2%) had visited antenatal health institutions at least once. Among the 331 (71.2%) mothers who delivered in a health institution, 145 (31.2%) of them had a spontaneous vaginal delivery (Table 2).

**Table 2:** Obstetric characteristics of women in South Tigray Zone towns, November, 2014

Factors	Frequency (%)	95% CI
Gravidity		
I	118 (25.4)	21.3–29.5
II–V	263 (56.6)	51.8–60.9
≥VI	84 (18.1)	14.6–21.5
Neonatal death		
No	334 (71.8)	67.7–75.9
Yes	131 (28.2)	24.1–32.3
Index ANC follow-up		
Yes	345 (74.2)	70.1–78.3
No	120 (25.8)	21.7–29.9
Actual place of delivery		
Home delivery	134 (28.8)	24.5–32.9
Institution delivery	331 (71.2)	67.1–75.5
Birth preparedness and complication readiness		
No	98 (21.1)	18.2–26.6
Yes	367 (78.9)	73.4–81.8
Duration of labour (hours)		
≤3	24 (5.2)	3.2–7.3
3–24	428 (92)	89.2–94.6
>24	13 (2.8)	1.3–4.3
Obstetric problems during pregnancy		
No	54 (11.6)	8.8–14.8
Yes	411 (88.4)	85.2–91.2
Mode of delivery in health institution (*n*=331)		
Spontaneous vaginal delivery	185 (55.9)	38.9–72.7
Assisted vaginal delivery	78 (23.8)	20.3–27.4
Caesarean section	62 (18.7)	16.1–21.3
Other (including foetal destruction)	6 (1.8)	1.4–3.4

### Missed opportunity for modern delivery practice

**Fig. 1:**
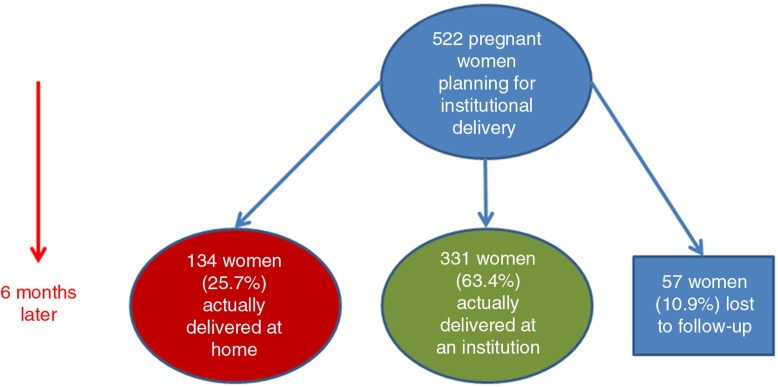
Planned and actual place of delivery among 522 pregnant women in South Tigray Zone towns, northern Ethiopia, November, 2014.

**Fig. 2:**
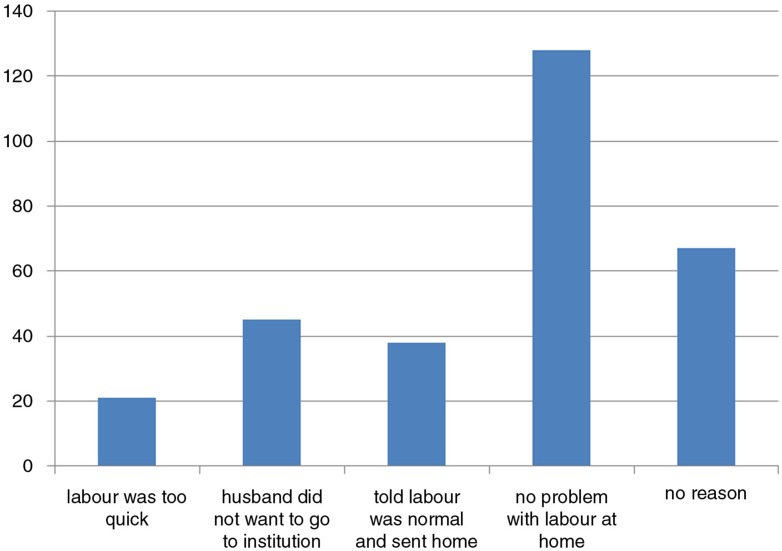
Reasons for home delivery, despite planned institutional delivery, among 134 pregnant women in South Tigray Zone towns, northern Ethiopia, November, 2014 (multiple reasons possible).

The study result revealed that 28.8% of the women missed their opportunity of modern delivery practice. Among 465 pregnant women who had planned to deliver their index pregnancy in a health institution, 331 (71.2%) actually delivered in a health institution (Fig. 1). The 134 (28.8%) mothers who delivered at home were asked the reasons why, when their planned place of delivery was in a health institution. The most frequently reported reasons were that no problem occurred during labour at home (128, 42.8%), followed by a change of decision (67, 22.4%) (Fig. 2).

### Factors associated with missed opportunity

A bivariate analysis was done to assess any association between independent variables and missed opportunity. After controlling the effect of other variables; marital and educational status, obstetric problems during pregnancy, birth preparedness and complication readiness status, ANC follow-up status and women's autonomy status were found to be significantly associated with missed opportunity of institutional delivery (*p*<0.05).

Single women were more than twice as likely to deliver at their home when compared with married women (AOR 2.42, 95% CI 1.16–5.06). Women who had no formal education were seven times more likely to deliver at their home compared to those who had attended college and above (AOR 6.95, 95% CI 2.24–21.6). Women who were not attending antenatal clinics during the index pregnancy were twice as likely to have unplanned home delivery compared to those who had at least one visit (AOR 2.24, 95% CI 1.27–3.94). Women who faced no obstetric problem during the index pregnancy were about three times more likely to have home delivery compared to those who faced at least one obstetric problem (AOR 2.96, 95% CI 1.47–5.97). Women who were not autonomous and could not make the final decision about their place of delivery themselves were twice as likely to have home delivery compared to those who were autonomous (AOR 2.20, 95% CI 1.29–3.75). Women with no birth preparedness and complication readiness were nearly five times more likely to deliver in their home compared to those who had already prepared and were ready for early management of complications (AOR 4.72, 95% CI 2.61–8.52) (Table 3).

**Table 3:** Bivariate and multivariate analyses of factors associated with institutional delivery service utilisation among pregnant mothers in South Tigray Zone towns, northern Ethiopia, November, 2014

Factors	Institutional delivery	Home delivery	Bivariate odds ratio (95% CI)	Adjusted odds ratio (95% CI)
Age of pregnant women				
15–19	25	8	Reference	
20–24	89	20	2.24 (0.96–0.22)	
25–29	108	28	3.18 (1.81–5.61)	
>30	109	78	2.76 (1.66–4.58)	
Educational status				
No formal education	112	56	7.10 (2.71–18.6)	6.95 (2.24–21.6)
Primary education	88	55	8.88 (3.37–23.4)	7.75 (2.45–24.5)
Secondary and high school	60	18	4.26 (1.49–12.16)	5.00 (1.44–17.4)
College and above	71	5	Reference	Reference
Marital status				
Married	39	29	Reference	Reference
Divorced	249	90	1.28 (0.62–2.64)	
Widowed	17	3	0.49 (0.14–1.71)	
Single	26	12	2.06 (1.20–3.52)	2.42 (1.16–5.06)
Gravidity				
1	93	25	Reference	
2–4	177	86	1.40 (0.73–2.69)	
≥5	61	23	1.81 (1.08–3.01)	
Neonatal death				
No	249	85	1.75 (1.14–2.69)	
Yes	82	49	Reference	
ANC follow-up				
No	68	52	2.45 (1.58–3.80)	2.24 (1.27–3.94)
Yes	263	82	Reference	Reference
Knowledge about institutional delivery service				
Poor	69	39	Reference	
Good	262	95	1.56 (0.99–2.46)	
Women making decisions				
No	81	57	2.29 (1.50–3.49)	2.20 (1.29–3.75)
Yes	250	77	Reference	Reference
Obstetric problems				
Yes	303	108	2.61 (1.46–4.64)	2.96 (1.47–5.97)
No	28	26	Reference	Reference
Birth preparedness and complication readiness				
No	48	50	3.51 (2.21–5.59)	4.72 (2.61–8.52)
Yes	283	84	Reference	Reference

## Discussion

Despite the relative accessibility of health institutions in urban areas, a significant number of urban resident pregnant women still delivered at home, contrary to their plans for institutional child birth. The current study revealed that 28.8% of urban resident pregnant women delivered at their home, even though they had planned for institutional delivery. The current finding is similar to a study done in Amhara region, which showed that among total urban resident women who had planned for institutional delivery, 20% delivered at their home (8). Our result is a little higher than in a Nepalese study, which showed among pregnant women who had planned for institutional delivery, only 16.3% delivered at their home. The difference could be due to stronger birth preparedness and complication readiness in Nepal (9).

The current finding shows a lower percentage of missed opportunity when compared to the results of a study done in Switzerland (10). The gap could be mainly due to the difference in the model of delivery service. In Ethiopia, home delivery follows model 1, where lay providers recognise complications occurring during delivery and the family organises access to a health institution with essential obstetric care (EOC). In developed countries such as Switzerland, home delivery is model 2, where labour is attended at home by professionals who recognise complications and family or provider organises referral to EOC (11).

From this prospective follow-up study, it was found that maternal education status is an important predictor of unplanned home delivery. Women with no formal education were seven times more likely to deliver at their home when compared to those who had attended college and above. This finding is consistent with studies conducted in different parts of Ethiopia; a study done in Bahir Dar town, Amhara regional state, showed that women with no formal education were 4.3 times more likely to have home delivery (12). On the other hand, many local studies have shown that women with higher educational levels were more likely than those who were illiterate to give birth in health institutions (5, 13, 14). Similar findings from studies in Sri Lanka and Bangladesh reported poor maternal education to be an important risk factor for home delivery (15, 16). This may be because uneducated women are not familiar with public institutions and feel uneasy about giving birth there. Additionally, it is obvious that modern education is a universally accepted strategy for improving preventive health behaviour in general.

Marital status was found to be associated with home birth. Single women were twice as likely to deliver at home compared with married ones. This finding is inconsistent with a study in Tigray regional state which showed unmarried women were twice as likely to deliver in a facility (17). A similar study in Nigeria reported that single women tended to have home deliveries compared with married ones (18). This could be explained by many pregnancies among single women being unintended and in young age groups. For unintended pregnancies, ANC follow-up is unlikely and family pressures may keep them at home because pregnancy before marriage is shameful for the family. Additionally married people, as measured by numerous health outcomes, are generally healthier than unmarried people (19).

Another finding of the present study was that not attending antenatal clinics is an important predictor of home delivery. Mothers who accessed no ANC during the index pregnancy were twice as likely to deliver at home compared to those who attended ANC.

This result is consistent with the findings from Bahir Dar town in Amhara region, where women with poor ANC attendance were 8.7 times more likely to deliver at their home (12). The current finding is also supported by a study in Sekela district which showed that women who attended ANC were four times more likely to deliver in health facilities than those who did not make ANC visits (20). The explanation for this may be that the absence of information as well as the experiences they might have had during the antenatal clinic visits might have influenced their decision to deliver at home. Another possible explanation may be that ANC follow-up itself is one kind of maternal service utilisation, so that factors which influence utilisation of ANC might have influenced institutional delivery service utilisation in a similar manner.

Women who did not face any obstetric problems during their index pregnancy were three times more likely to deliver at home compared with those who had at least one obstetric problem. This result is consistent with studies done in parts of Ethiopia: absence of obstetric complications during pregnancy and child birth was the most frequently reported reason for home delivery (21). According to a study done in Debre Markos town, women who had faced at least one obstetric complication during pregnancy were three times more likely to deliver in health institutions (8).

Studies from Kenya and Nepal showed that most women who had obstetric complications sought professional help. On the other hand, the main reason for home delivery was the absence of complications or problems during labour at home (22, 23). This may be due to the fact that women with such complications during pregnancy develop a sense of susceptibility to severe childbirth complications and are more likely to seek help from health professionals (8).

Women who were disempowered from choosing their place of delivery were twice as likely to have home deliveries compared with those who had full autonomy. This finding is consistent with studies done in Bangladesh and Ghana on determinants of institutional delivery. About 45% of women with high autonomy compared to 37% of women with low autonomy had their last delivery at a health institution. Ghanaian women with higher autonomy were 1.5 times more likely to use medical institutions for delivery than those with lower autonomy (24, 25). The current result is also congruent with other studies done in different parts of Ethiopia where mothers with full power of decision making are more likely to have modern delivery practices (20, 21). Pregnant women who were insufficiently prepared for childbirth and possible complications (BPACR) were about 3.5 times more likely to have home deliveries. The same concept was also reported from research done in south Ethiopia (26, 27). The latter result is also consistent with the studies done among slum women of Indore city in India which showed that skilled attendance during delivery was three times higher in well-prepared mothers compared to less-prepared mothers (28). This could be explained by better birth preparedness being considered as an intervention fostering preventive behaviour and influencing other socio-economic and cultural barriers, thus encouraging the use of health facilities.

## Conclusions

A significant proportion of urban resident pregnant women in Ethiopia still miss the opportunity to access modern delivery practices in health facilities. Reasons given by mothers for giving birth at home, against their plan, included no problems during labour at home; opposition from husbands, labour was urgent, no particular reason and misunderstandings after being sent home during the latent phase of labour.

Educational status, ANC compliance, obstetric complications during pregnancy, birth preparedness and complication readiness, and women's autonomy were important predictors of institutional delivery. This provides important pointers to sub-groups of women who might be particularly targeted with messages about facility delivery during their pregnancies.
